# Swiss Sepsis National Action Plan: A coordinated national action plan to stop sepsis-related preventable deaths and to improve the support of people affected by sepsis in Switzerland

**DOI:** 10.3389/fmed.2023.1114546

**Published:** 2023-02-20

**Authors:** Luregn J. Schlapbach, Elisa A. Zimmermann, Sylvain Meylan, Martin Stocker, Peter M. Suter, Stephan M. Jakob, on behalf of the Swiss Sepsis National Action Plan Working Group

**Affiliations:** ^1^Department of Intensive Care and Neonatology, and Children’s Research Center, University Children's Hospital Zurich, Zurich, Switzerland; ^2^Child Health Research Centre, The University of Queensland, and Paediatric Intensive Care Unit, Queensland Children’s Hospital, Brisbane, QL, Australia; ^3^Infectious Diseases Service, Department of Medicine, Lausanne University Hospital and University of Lausanne, Lausanne, Switzerland; ^4^Department of Pediatrics, Children's Hospital Lucerne, Lucerne, Switzerland; ^5^University of Geneva, Geneva, Switzerland; ^6^Department of Intensive Care Medicine, Bern University Hospital, University of Bern, Bern, Switzerland

**Keywords:** sepsis, National Action Plan, awareness, prevention, support sepsis survivor, recommendation, sepsis standard

## Abstract

**Background:**

Sepsis is a devastating disease which causes yearly over 10 million deaths worldwide. In 2017, the World Health Organization (WHO) issued a resolution prompting member states to improve the prevention, recognition, and management of sepsis. The 2021 European Sepsis Report revealed that—contrary to other European countries—Switzerland had not yet actioned the sepsis resolution.

**Methods:**

A panel of experts convened at a policy workshop to address how to improve awareness, prevention, and treatment of sepsis in Switzerland. Goal of the workshop was to formulate a set of consensus recommendations toward creating a Swiss Sepsis National Action Plan (SSNAP). In a first part, stakeholders presented existing international sepsis quality improvement programs and national health programs relevant for sepsis. Thereafter, the participants were allocated into three working groups to identify opportunities, barriers, and solutions on (i) prevention and awareness, (ii) early detection and treatment, and (iii) support for sepsis survivors. Finally, the entire panel summarized the findings from the working groups and identified priorities and strategies for the SSNAP. All discussions during the workshop were transcribed into the present document. All workshop participants and key experts reviewed the document.

**Results:**

The panel formulated 14 recommendations to address sepsis in Switzerland. These focused on four domains, including (i) raising awareness in the community, (ii) improving healthcare workforce training on sepsis recognition and sepsis management; (iii) establishing standards for rapid detection, treatment and follow-up in sepsis patients across all age groups; and (iv) promoting sepsis research with particular focus on diagnostic and interventional trials.

**Conclusion:**

There is urgency to tackle sepsis. Switzerland has a unique opportunity to leverage from lessons learnt during the COVID-19 pandemic to address sepsis as the major infection-related threat to society. This report details consensus recommendations, the rationale thereof, and key discussion points made by the stakeholders on the workshop day. The report presents a coordinated national action plan to prevent, measure, and sustainably reduce the personal, financial and societal burden, death and disability arising from sepsis in Switzerland.

## Introduction

### The need for a sepsis action plan in Switzerland

**What is Sepsis? What is the burden of Sepsis? Why do we need a Sepsis National Action Plan in Switzerland?**

Sepsis arises when the body’s response to an infection injuries its own tissues and organs (1). It may lead to shock, multi-organ failure, and death, especially if not recognized early and treated promptly. Most commonly, sepsis is caused by bacterial infections which can be acquired in the community or in a healthcare setting (so called nosocomial, or healthcare-associated sepsis). Other pathogens, including viruses and fungi, can result in sepsis too. In fact, many patients with COVID-19 manifest sepsis (2). Importantly, sepsis represents the common pathway of severe organ failure and death resulting from most infectious diseases. While patients at the extremes of age (neonates, children, and elderly people) are most vulnerable to sepsis, sepsis is a major cause of mortality and morbidity across all age groups.

In Switzerland, data from 2017 which were obtained through national disease coding datasets, indicate that every year over 19,000 persons suffer from sepsis (3), and almost 3,500 patients will die because of sepsis every year. Of those who survive, it is estimated that up to half are left with a disability or impaired function (4). Nevertheless, these numbers likely substantially underestimate the true burden of sepsis, since reports from other countries have shown that sepsis cases and sepsis deaths are often attributed to the underlying infection and are therefore not accurately counted (5, 6). In comparison, sepsis thus kills more patients than leading cancer groups (annual deaths in Switzerland 2014–2018 were for lung cancer: 3,300; large bowel cancer: 1,700; breast cancer: 1,410; Prostate cancer: 1,400).^1^

Exact costs resulting from sepsis in Switzerland are unknown. A previous study using data from 1998 to 2000 observed an average direct cost of CHF 41,790 (standard deviation CHF 33,222) per sepsis case, and estimated annual costs of CHF 493 to 1,199 million per year in Switzerland (7). Importantly, true total societal costs related to sepsis are magnified several folds: first, there are post-sepsis costs associated with new impairments and new healthcare requirements after sepsis. In a large national German study, average health costs of € 29,088 (standard deviation € 44,195) per sepsis survivor have been calculated for the first 3 years post sepsis (8). Second, indirect costs relate to life years lost, reduced, or lost work capacity of patients, long-term cognitive, physical, or mental impairments affecting professional performance, as well as spouses, parents, and children taking carer roles with associated reduced professional and economic performance. As post-sepsis sequelae may persist life-long, the combined effect on societal costs is enormous.

Sepsis affects worldwide 49 million humans every year resulting in 11 million deaths (3). It has been therefore declared a priority for global health by the World Health Assembly at the World Health Organization (WHO) in 2017 (9). The WHA70.7 resolution (Sepsis resolution, 2017) called the member states to take action in developing and implementing national strategies to improve prevention, diagnosis and management of sepsis. Five years after this resolution, many European countries have developed coordinated programs in collaboration with governments, professionals and patient-advocacy groups. In 2021, the European Sepsis Alliance published the European Sepsis Report 2021,^2^ showcasing measures undertaken by several European countries. Switzerland is not included into this report, since until now Switzerland has lacked a coordinated approach to tackle sepsis.

The burden imposed by sepsis in Switzerland contrasts with the lack in public awareness, insufficient institutional efforts, as well as absence of national coordination and monitoring to reduce the impact of sepsis. Sepsis has often been called a disease of systematic failure to learn. Root-cause-analyzes of patients who die of sepsis commonly reveal reoccurring patterns of delayed presentation due to lack of awareness, delayed recognition by healthcare staff, and missed opportunities for effective interventions once sepsis is recognized (10). In addition, survivors and family members are often left poorly informed about sepsis and its long-term sequelae which are not appropriately addressed by existing support structures. Other healthcare systems have summarized these challenges unique to sepsis as the combined effect of a lack of (11):

– Awareness and education of the public and healthcare workforce
– Standards and pathways for sepsis recognition and treatment
– Follow-up systems for survivor and family support and rehabilitation

In summary, sepsis is a life-threatening condition and is accountable for a major proportion of potentially preventable mortality and morbidity in Switzerland. The aim of the Swiss Sepsis National Action Plan (SSNAP) is to stop preventable deaths and to support people affected by sepsis. Specifically, the SSNAP outlines strategies and priorities in order to realize the goals of the recent World Sepsis 2030 declaration, aiming to develop solutions designed to meet the needs of the Swiss population and healthcare system^3^

1. To improve public awareness of sepsis.
2. To decrease sepsis incidence across all age groups.
3. To improve and sustain 3 pillars of infection management which are joint at government policy level:

- infection prevention
- antimicrobial stewardship (AMS)
- sepsis recognition and management

4. To increase sepsis survival across all age groups thanks to the implementation of rapid recognition and response standards of care.

5. To ensure that sepsis survivors can access support & rehabilitation services, allowing survivors and families to regain social and professional integration faster.

### What can we learn from the experience in other countries?

The experience from other countries or regions, such as Australia, United Kingdom, and the United States consistently demonstrates that coordinated actions against sepsis can save thousands of lives, improve the outcomes for sepsis survivors, and have a high chance to be cost saving for the healthcare system (12, 13). Evidence from the State of New York, which introduced in 2013 a mandate for evidence-based sepsis protocols for all healthcare services, shows that the measure was associated with an adjusted absolute mortality reduction of 3.2% (95%-confidence interval 1.0% to 5.4%, *p* = 0.004) compared to states which did not introduce a sepsis mandate (14–16).

The key pillars of different sepsis quality improvement programs are remarkably similar when comparing countries and healthcare services who have successfully implemented sepsis campaigns. They are characterized by a comprehensive approach to integrate traditional healthcare improvement methodology with coordinated public health and policy measures:

1. **Coordinated policy approach:** involvement of professional bodies and stakeholders across government, academia, community, hospital, and general practice settings.
2. **Implementing standards for healthcare professionals**: development of protocols for recognition, treatment, and follow-up of sepsis, systematic education of the healthcare workforce on sepsis, standardized clinical data collection and registries to measure impact.
3. **Public awareness**: increasing public knowledge and awareness about sepsis, use of media and advertisements through a targeted campaign.
4. **Cooperation and synergies**: inclusion of multidisciplinary experts, patient and public involvement (PPI), as well as strategic collaboration with large-scale research programs.

### Putting sepsis into the Swiss public health context

Switzerland as one of the wealthiest countries in the world has a highly developed healthcare system, with a high density of medical services, hospitals and academic facilities. Health insurance is mandatory for all people of all ages living in Switzerland. The majority of healthcare is delivered through the public system which is covered by the mandatory healthcare insurance; with additional optional insurance available for private cover (17). The Federal Office of Public Health (FOPH – BAG) has the responsibility to protect public health, develop Swiss health policy and ensure that the country has an efficient healthcare system.^3^ The Division of Communicable Diseases monitors infectious diseases and regularly reports on the epidemiological situation while implementing prevention and control strategies. Although by 2022 no specific actions to fight sepsis have been started at the FOPH, several important strategies have been conducted which aim at preventing and controlling infectious diseases and which thereby contribute to the prevention and treatment of sepsis:

- The **Swiss NOSO Strategy** was ordered by the Federal Council in 2016 and aims at improving patient safety by reducing healthcare associated infections in the inpatient setting. The NOSO strategy sustains several projects which interface with other existing strategies and has as common goal the reduction of hospital and nursing home infections.^4^
- The **National Vaccination Strategy (NVS)** aims to protect the population adequately against vaccine-preventable diseases. This strategy was formulated in 2012, and in 2017, a national action plan was implemented. A second implementation phase is planned for 2024–2028.^5^
- The **Antibiotic Resistance Strategy (StAR)** pursues the overarching goal to ensure the efficacy of antibiotics for humans and animals in the long term and to help standardize the use of antibiotics and reduce inappropriate consumption. The strategy has been elaborated in 2013–2015 in cooperation with different Federal Authorities: the Federal Office of Public Health (FOPH), the Federal Food Safety and Veterinary Office (FSVO), the Federal Office for Agriculture (FOAG), and the Federal Office for the Environment (FOEN). In 2013 the first joint national report on comprehensive monitoring of antibiotic resistance and antibiotic use in human and in veterinary medicine was released. In 2016 the first Swiss antibiotic resistance report was published.^6^

These existing strategies should cross-fertilize with the implementation of the SSNAP. Fundamental to the realization of new strategies focusing on quality improvement is the Federal Quality Commission (FQC), which is an independent extra-parliamentary expert commission. It was appointed by the Federal Council for a period of 4 years (currently until 2024). The financing of the costs of FQC for its operation is ensured by the Confederation, the cantons and the insurers in equal parts. The FQC supports the Federal Council in quality development in medical service provision within the framework of the Federal Health Insurance Act. Moreover, it advises and coordinates the various actors and supports financially national and regional quality development projects.

Finally, Swiss institutions have participated in internationally highly recognized research on sepsis in children and adults. For example, the Swiss National Science Foundation (SNSF) funded Swiss Pediatric Sepsis Study investigated the epidemiology, as well as the genetic background of sepsis in children during 2011–2015 (18). Swiss experts were key to formulate a roadmap for sepsis research (19). More recently, the Swiss Personalized Health Network (SPHN) and the Personalized Health Related Technologies strategic focus area of the ETH Domain (PHRT) have funded a national data stream focusing on sepsis in adult ICU patients (20).

### Lessons learned from the COVID-19 pandemic

The emergence of the COVID-19 pandemic has presented the world with one of the most serious health threats in living memory. Also unprecedented was the global response to the pandemic: policymakers, health care providers, industry, and the scientific community have come together and enabled the development of robust evidence for best treatment and novel vaccines within a record time. Simultaneously, the public awareness about the vulnerability of the human species for infectious diseases, and the role of organ dysfunction and ICU support dramatically increased. Moreover, the comprehensive approach included the rapidly emerging evidence of COVID-19 associated long term sequelae and initiated the establishment of post-rehabilitation support strategies (21).

Within the framework of a federal Swiss healthcare system, comprehensive and integrated approaches across the country resulted in reliable measures of disease burden, effective interventions, and highly effective research and public health responses.

Early recognition of new variants and viral lineages was critical during the pandemic. The molecular epidemiological monitoring coordinated *via* the Federal Office of Public Health, the National Reference Center for Novel Viruses (CRIVE), and the Swiss Pathogen Surveillance Platform (SPSP^7^) was tremendously helpful, and achieved to sequence more than 140′000 viral genomes. The molecular surveillance of antibiotic drugs resistance and hypervirulent bacterial strains, as well as exchange of pathogen genomic data through platforms such as SPSP, will be very important for Sepsis. This will further support the development of new rapid diagnostics and research.

The pandemic has shown how important a coordinated response is to tackle severe infectious diseases and has helped to create more effective partnerships across hospitals, academia, government, and the public. COVID-19 patients present common manifestations that characterize sepsis (22), and many patients with COVID-19 ultimately develop sepsis (23). The SSNAP should consider the lessons learnt from the pandemic, including creation of public awareness, preventive and community interventions, agile data-driven management of the disease, and rigorous implementation of best practice at all hospitals for the diagnosis, management, and after-discharge care. Let us do the same for sepsis!

### Barriers to quality improvement for patients with sepsis in Switzerland

The SSNAP panel of experts identified a number of barriers and obstacles which are key to consider when designing strategies suitable for the Swiss context:

- **Lack of public awareness of sepsis, and lack of public understanding of the term “sepsis.”** Contrary to diseases such as “stroke,” “cardiac infarction,” “cancer,” or “AIDS,” the term “sepsis” appears to be little used in the public. Surveys in Germany and Australia indicated that less than half of adults had basic knowledge of sepsis. While we lack exact data on sepsis awareness in the Swiss population, these studies suggest that it may be low. In addition, the link between vaccination campaigns and sepsis prevention, or between COVID-19 and sepsis is usually absent in the public perception. Furthermore, sepsis as a concept of dysregulated host response to infection leading to life-threatening organ dysfunction may be complex to grasp in lay terms, implying a need for professionally conducted public awareness campaigns ensuring common simple language.
- **Limited training of the healthcare workforce on sepsis, and on the importance of quality improvement.** Surveys in Switzerland as well as in other high income countries indicate that often healthcare staff, and even medical and nursing students are insufficiently trained in sepsis prevention, recognition and management (24–26).
- **Lack of a national database capturing sepsis in Switzerland.** Contrary to many other diseases for which well-established national registries exist, there is no routine data collection for patients with sepsis and it is likely that diagnostic coding may be insufficiently accurate. This hinders reliable assessment of sepsis burden, rapid feedback to clinicians and stakeholders in relation to performance metrics, as well as robust measurement of the impact of sepsis quality improvement.
- **Sepsis as an inherently multidisciplinary disease in a multi-siloed healthcare system.** Sepsis can affect any patients of any age at any facility and therefore does not “belong to a single discipline.” Correspondingly, individual expertise around sepsis may vary, and patients with sepsis may be disproportionally affected by fragmented and siloed healthcare.
- **Lack of a standard pathway to facilitate the screening, recognition, treatment, and follow-up of patients with sepsis in Switzerland**. While many hospitals have sepsis guidelines, these are not usually implemented systematically, nor monitored regularly. Similarly, there are no established follow-up support systems.
- **Traditional culture of doctor-determined, hierarchical healthcare.** Many initiatives have shown the importance that any healthcare worker, irrespective of profession or hierarchical status, is empowered to action timely recognition and treatment for sepsis. Systematic quality improvement for sepsis thus goes hand in hand with safety culture developments, such as “Speaking up for safety.”
- **Lack of standardized systems for the recognition of deteriorating patients in Switzerland.** Contrary to many, in particular Anglo-Saxon healthcare settings, rapid response teams or Early Warning Scores (EWS) are not widely implemented in Switzerland (27). This potentially impacts on the capacity to early recognize deteriorating patients. Sepsis is one of the leading causes of in-hospital patient deterioration.
- **Insufficient compliance with evidence-based measures shown to potentially prevent sepsis.** Routine measures of hand hygiene, and compliance with central line insertion bundles are not performed at frequent intervals across all hospitals in Switzerland, nor are there transparent inter-facility monitoring data available for these internationally established benchmarks.
- **Federalism and lack of a centralized body monitoring and benchmarking quality in healthcare.** Until recently, data on the quality of the Swiss healthcare system was hard to obtain for the public. This may in part reflect the cantonal system, which traditionally may have interfered with national benchmarking. The report on the Quality in the Swiss Healthcare System observed that a number of quality control systems, as well as quality improvement initiatives were less developed compared to other high income countries. The report recommended actions to improve the training of the healthcare workforce in evidence-based high quality care such as handovers, recognition of deteriorating patients, team work and simulation.
- **Lack of sepsis-specific mandated quality indicators governing the accreditation of healthcare professionals, as well as healthcare institutions.** The Swiss National Association for Quality Development in Hospitals and Clinics (ANQ) captures postoperative infections, not sepsis specifically, as a standardized quality indicator. In addition, at present there are no formal requirements from either government, policy, nor any of the medical bodies (FMH or societies) mandating sepsis-specific quality indicators.
- **Potential perception of sepsis quality improvement opposing strategies to reduce use of antibiotics.** The use of timely antibiotics is the single most effective measure in the treatment of sepsis. Accordingly, there is potential concern that sepsis initiatives may promote indiscriminate use of broad-spectrum antibiotics, which may promote antimicrobial resistance (28, 29). Therefore, sepsis quality improvement should aim to reinforce the importance of AMS with the goal that the right patients receive the right antibiotics at the right time.
- **Lack of patient and family organizations specific to sepsis.** Contrary to several patient cohorts, at present there are no specific patient survivor or family support groups for those affected by sepsis in Switzerland.
- **Limited tradition in pragmatic interventional, quality improvement, and healthcare service research.** Contrary to Switzerland’s outstanding reputation in the field of basic science, research institutions such as SNSF have traditionally given less weight to healthcare service research investigating the implementation and efficacy of common interventions to common diseases such as sepsis. While this field of research is recently receiving more attention, the funding allocated to such areas, and to sepsis in particular remains substantially less compared to for example the National Institute for Health and Care Research (NIHR) scheme in the United Kingdom.

Importantly, addressing these barriers in the context of sepsis in Switzerland may yield desirable collateral benefits for other diseases, for example through the improved recognition of deteriorating patients in siloed healthcare settings and improved preparedness for future pandemics.

## Methods

A panel of more than 50 experts convened at a policy one-day workshop to address the pressing need to improve awareness, prevention, and treatment of sepsis in Switzerland. The goal of the day was to formulate a set of consensus recommendations toward creating a Swiss Sepsis National Action Plan (SSNAP). The workshop was professionally facilitated and took place on the 10th of June 2022 in Berne, Switzerland. The panel included key stakeholders from across the health sector from different disciplines and professions, members of the FOPH and of the FQC, academic professionals, and sepsis survivors from different Swiss regions (30).

The workshop started with talks from international speakers that summarized the experiences from sepsis quality improvement programs in the UK, US, Germany, and Australia. National stakeholders then gave an overview on existing health programs in Switzerland and their relevance for sepsis. Thereafter, the participants were allocated into three working groups to identify opportunities, barriers, and solutions on the key domains: Prevention and awareness, early detection and treatment, and support for sepsis survivors.

Each working group was led by a facilitator. The groups independently explored the challenges pertinent to their allocated domain, identified correctable gaps in current services, and potential solutions for a whole of society and whole of health system approach. At the end of the workshop, the entire panel summarized the findings from the working groups and identified priorities and strategies for the SSNAP. All discussions during the workshop were recorded, and then transcribed into the present document. Recommendations were sent back to the whole panel, who indicated if they agreed with the formulation, or requested modifications. Finally, the full SSNAP document was circulated for further input among workshop participants and key experts who had been unable to attend the workshop.

The SSNAP report has been professionally translated into English, German, Italian, and French. All reports have been published at the World Sepsis Day, the 13th of September 2022, on the Swiss Society of Intensive Care Medicine homepage (30).

## Results

**Figure 1:**
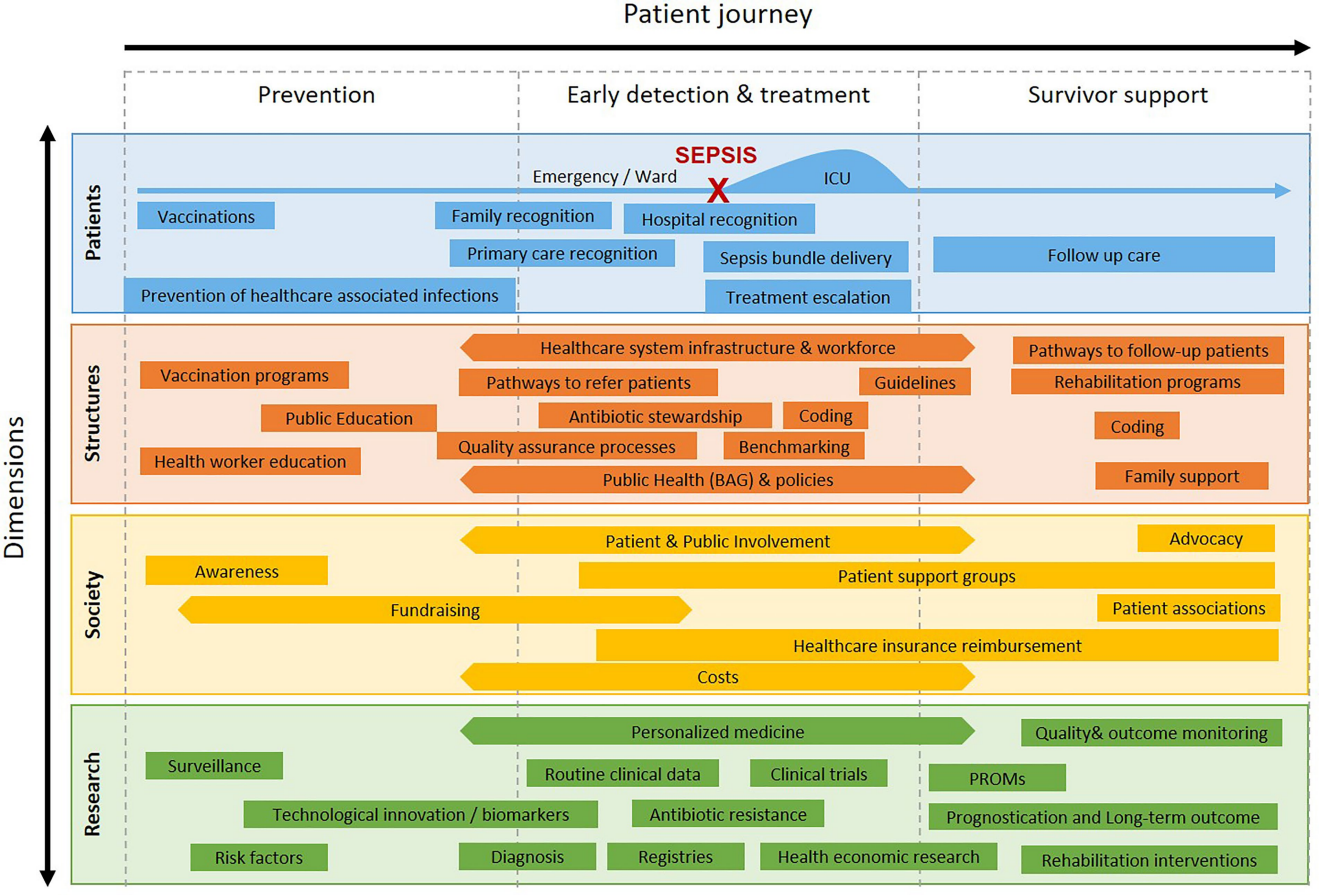
Overview on key topics identified by the Swiss Sepsis National Action Plan workshop, across the patient journey in relation to patient, structure, society, and research dimensions.

Based on a collaborative and solution-focused discussion, key recommendations were developed at the SSNAP workshop (Table 1). The focus of the discussion resided on the three domains of “prevention and awareness,” “early detection and treatment,” and “survivor support.” These three domains were analyzed across different dimensions (Figure 1), including patients, structures (healthcare system and policy organizations), society (population), and research. For each dimension across the patient journey, key topics were identified and addressed by the SSNAP (Figure 1).

**Table 1:** Key recommendations.

**Recommendation 1:** **Launch a national sepsis awareness and education campaign targeting the public, as well as the healthcare workforce.**	
Recommendation 1a	Improve and maintain the training of the healthcare workforce in sepsis including students, and hospital-, and community-based healthcare workers.
Recommendation 1b	Design and conduct a public sepsis awareness campaign.
Recommendation 1c	Improve the education and compliance with evidence-based measures to prevent healthcare-associated infections, strengthen routine reporting on hospital associated infections across institutions, and support existing strategies and bodies involved in this field, in particular Swissnoso.
Recommendation 1d	Strengthen existing infection prevention strategies including through vaccinations with particular reference to their potential to prevent sepsis.
**Recommendation 2:** **Establish and implement a minimal national standard for the detection, treatment, and follow-up of sepsis.**	
Recommendation 2a	Define a minimal (“core”) national standard for the detection and treatment of sepsis.
Recommendation 2b	Implement sepsis pathways for emergency department and in-hospital patients in Swiss hospitals.
Recommendation 2c	Include antimicrobial stewardship (AMS) in the design, training, and evaluation of sepsis pathway implementation.
Recommendation 2d	Establish a national sepsis registry to monitor short- and long-term disease burden and benchmark practice.
Recommendation 2e	Include sepsis incidence, treatment, and outcomes as quality indicators in healthcare reporting.
**Recommendation 3:** **Establish and implement support systems for sepsis survivors and for families affected by sepsis.**	
Recommendation 3a	Develop information and education materials on long-term outcomes after sepsis to educate patients and healthcare workers.
Recommendation 3b	Design follow-up and rehabilitation pathways for sepsis patients building on existing structures including hospital care, rehabilitation services, allied health, and family doctors, which link the hospital to post-discharge care.
Recommendation 3c	Establish support structures for families affected by sepsis including sepsis specific patient interest groups.
**Recommendation 4:** **Promote national sepsis research including translational, healthcare service, and basic science research.**	
Recommendation 4a	Fund a national sepsis research program (NRP).
Recommendation 4b	Promote the participation of Swiss institutions in national and international diagnostic and interventional sepsis trials, and support the creation of trial platforms for sepsis patients.

### Prevention and awareness

#### Recommendation 1: Launch a national sepsis awareness and education campaign targeting the public, as well as the healthcare workforce

**Recommendation 1a**: Improve and maintain the training of the healthcare workforce in sepsis including students, and hospital-, and community-based healthcare workers.
**Recommendation 1b**: Design and conduct a public sepsis awareness campaign.
**Recommendation 1c**: Improve the education of and compliance with evidence-based measures to prevent healthcare-associated infections, strengthen routine reporting on hospital associated infections across institutions, and support existing strategies and bodies involved in this field, in particular Swissnoso.
**Recommendation 1d**: Strengthen existing infection prevention strategies including through vaccinations with particular reference to their potential to prevent sepsis.

#### Specific comments and specific strategies to consider

- Conduct public surveys to assess the knowledge and perception of sepsis, as well as to evaluate the effect of awareness campaigns.
- Deliver a consistent message in public awareness and education strategies to enable a common language and framework: what is sepsis, why is sepsis an emergency, what can you do to reduce the impact of sepsis.
- Professionally design and conduct public information campaigns on sepsis. For example, the FOPH-BAG led campaign on HIV (a disease which infected at most just over 1,000 patients per year in Switzerland) has been highly visible, effective, and sustainable. Therefore, the FOPH-BAG seems ideally suited to lead such a campaign. Support from health insurance companies and pension funds should be sought.
- Ensure that campaigns amplify key messages– for example, vaccination campaigns should highlight the impact of vaccines on reducing sepsis.
- Target healthcare focused campaigns not only to hospital workers, but also to family doctors as a first-line contact for most patients; and also to pharmacies, dentists, physiotherapists, paramedics, psychologists, Spitex/CMS, and nursing home staff.
- Update medical university and nursing school curricula to ensure contemporary data on sepsis are covered, including prevention, recognition, treatment, and follow-up of sepsis; as well as state-of-the-art information on the importance and impact of sepsis quality improvement.
- Give structured education to mothers on signs of neonatal sepsis, as this has been shown to reduce mortality in low resource settings. Systematic education of patients and families has helped drop mortality in oncologic patients with fever in neutropenia in the past decades. Similar strategies are likely to improve timely recognition of sepsis, for example using leaflets, newsletters, and checklists for routine health appointments, such as information given in child development checkups (like the “Gesundheitsheft” of the Swiss Society of Pediatrics).
- Include sepsis, and sepsis signs in secondary and high school curricula.
- Inform patients discharged from hospital or ambulatory care on how to recognize sepsis, including patients where a milder infection is diagnosed, to enhance prevention and early recognition.
- Improve education of medical and nursing students and staff on evidence-based measures to reduce health-care associated infections.
- Improve frequency and transparency of reporting of health-care associated infections, facilitated by Swissnoso.
- Incentivize hospitals and healthcare providers to improve compliance with evidence-based measures to reduce health-care associated infections.

### Early detection and treatment

#### Recommendation 2: Establish and implement a minimal national standard for the detection, treatment, and follow-up of sepsis

**Recommendation 2a**: Define a minimal (“core”) national standard for the detection and treatment of sepsis.
**Recommendation 2b**: Implement sepsis pathways for emergency department and in-hospital patients in Swiss hospitals.
**Recommendation 2c**: Include antimicrobial stewardship (AMS) in the design, training, and evaluation of sepsis pathway implementation.
**Recommendation 2d**: Establish a national sepsis registry to monitor short- and long-term disease burden and benchmark practice.
**Recommendation 2e**: Include sepsis incidence, treatment, and outcomes as quality indicators in healthcare reporting.

#### Specific comments and specific strategies to consider

- Define elements of a “core” minimal standard for sepsis recognition and treatment using a multidisciplinary Swiss working group. Recently, the Australian Commission on Quality and Safety in Healthcare has established best practice recommendations defining a national standard for the recognition and treatment of sepsis through extensive systematic reviews^8^. This standard was released on June 2022 and could be adapted to the Swiss context to speed up the process and save resources.
- Consider that no single tool or lab marker will be perfect or sufficient on its own; therefore, a focus on key messages aiming to assess whether a patient is becoming critically unwell in the setting of a suspected infection (“Red Flags”) is recommended.
- Develop sepsis-specific pathways for emergency department and in-hospital patients which cover the patient journey (Figure 1) from screening and recognition, to treatment and escalation, to discharge and post sepsis care. This will allow the creation of a “core” or model pathway, which can then be locally adapted.
- Train all healthcare professions, and include routine mandatory “eLearnings” to enable uptake, compliance, and sustainability of the pathways. Such learning modules would benefit from having a central repository platform which can be easily shared across Swiss institutions to save resources at local facilities. Training needs to be adapted to the age of the patient.
- Empower families and healthcare staff to raise the question “Could this be sepsis?” through targeted public information strategies. Consider providing gender specific communication and education given that many carers are mothers and wives.
- Collaborate with “Speaking Up” campaigns to include sepsis as a common condition involved in causing patient deterioration. Empowerment of every healthcare team member, as well as family members, to support sepsis recognition.
- Integrate first line points of contact for many out of hospital patients such as pharmacies, phone/tele-advice, insurers, and Spitex/CMS.
- Seek coordination with institutional systems designed to assist in the recognition and treatment of deteriorating patients in-hospital, such as rapid response teams (RRT), hospital code teams, critical patient review processes. Facilitate access to sepsis-specific information and protocols which can be titrated to the needs of each institution. Enhance the message that “sepsis is an emergency,” “every minute counts,” “acting fast can save lives.”
- Learn from coordinated rapid escalation pathways for stroke, trauma, myocardial infarction – which are time critical conditions similar to sepsis. Adapt such systems to rapid sepsis care.
- Evaluate the use of Early Warning Tools to recognize deteriorating in-patients. Ensure sepsis is highlighted as a common cause of deterioration, and that improved recognition of sepsis goes hand in hand with improved recognition of any patient deterioration.
- Where feasible, develop, test and implement digital resources assisting in sepsis screening and recognition, and capture sepsis treatment and outcomes. With the increasing digitalization of healthcare in Switzerland, such approaches have huge potential to provide representative data, reduce manual data collection, and speed up evaluation and feedback. Furthermore, digitally supported sepsis recognition may enhance timely treatment. Attention to alarm fatigue and uptake of digitalization in real practice is key.
- Develop lay information for patients and family members affected by sepsis informing them on what sepsis is, what they may experience, and what happens after discharge.
- Provide information to patients and families on how to recognize sepsis in case of deterioration when sepsis has been ruled out and patients are not admitted to hospital. This may contribute to raising public awareness.
- Enhance reliable and structured handover of information related to the patient to improve sepsis care further; for example, when transferring a patient from the emergency department to an in-patient ward.
- Create joint working groups of the SSNAP and the national StAR initiative, as well as Swissnoso to maximize effectiveness of coordinated recommendations and interventions. Healthcare workforce training needs to incorporate AMS education. Similarly, sepsis quality improvement initiatives should monitor compliance with AMS standards.
- Harmonize the national sepsis registry to be created with internationally available sepsis databases to reduce efforts to setup a registry and enable future learnings and comparisons. The registry should benefit from the expertise acquired in other registries in Switzerland, such as the cancer registry. Definition of key quality indicators is required across sepsis incidence, treatment, and outcome (mortality, ICU and hospital length of stay). Enable modular expansion of the registry to facilitate data collection in the setting of institutional quality improvement initiatives (such as additional process, balancing, or outcome measures).
- Enable harmonized extraction of routine healthcare data for the sepsis registry. The Swiss Personalized Health Network datastreams and interoperability framework would be ideally suited for this purpose and could support both quality control, benchmarking, as well as research.
- Use a pragmatic and standardized approach consistent with Sepsis-3 criteria for adults (and adapted for children) aligned with Swiss Diagnosis Related Groups (SwissDRG).

### Sepsis survivor support

#### Recommendation 3: Establish and implement support systems for sepsis survivors and for families affected by sepsis

**Recommendation 3a**: Develop information and education materials on long-term outcomes after sepsis to educate patients and healthcare workers.

**Recommendation 3b**: Design follow-up and rehabilitation pathways for sepsis patients building on existing structures including hospital care, rehabilitation services, allied health, and family doctors, which link the hospital to post-discharge care.

**Recommendation 3c**: Establish support structures for families affected by sepsis including sepsis specific patient interest groups.

#### Specific comments and specific strategies to consider

- Define elements essential to discharge planning, follow-up, and rehabilitation efforts as part of the national minimal standard for sepsis management.
- Develop structured screening for post sepsis syndrome as part of routine post discharge follow-up in combination with experts in general practice, rehabilitation, mental health, as well as allied health. Identify a post discharge main point of contact (“owner”/“case-manager” of the post discharge process), and ensure strong ties with the general practitioners who often are key points of contact for the patients.
- Leverage from discharge planning and rehabilitation pathways, which have been successfully established in other diseases such as myocardial infraction, stroke, or traumatic brain injury.
- Plan after hospital care already during the hospital stay, e.g., assessment of need for post-discharge support. Assess need for support in different domains (medical, daily living, financial, educational) routinely, for example through a pre-discharge checklist. Consider socioeconomic and cultural factors.
- Prepare lay information brochures on post-sepsis syndrome accessible to patients, families, and the public, including school teachers. Many patients with sepsis leaving the hospital report that they did not understand what happened to them.
- Educate the health workforce, including allied health, on post sepsis syndrome signs and symptoms, interventions and its importance.
- Fund professional support of sepsis survivor groups including social worker and psychology expertise in partnership with sepsis peer support groups.
- Provide early access to rehabilitation interventions.
- Include long-term outcomes in the national sepsis registry. Establish patient-reported outcome measures (PROM) as well as data linkage on long-term outcomes of sepsis patients where feasible.
- Acknowledgement and recognition of post sepsis syndrome as a relevant entity by the relevant stakeholders, including insurances.
- Ensure reimbursement of rehabilitation efforts related to post-sepsis syndrome.

### Research

#### Recommendation 4: Promote national sepsis research including translational, healthcare service, and basic science research

**Recommendation 4a**: Fund a national sepsis research program (NRP).
**Recommendation 4b**: Promote the participation of Swiss institutions in national and international diagnostic and interventional sepsis trials, and support the creation of trial platforms for sepsis patients.

#### Specific comments and specific strategies to consider

- Prioritize sepsis research through SERI (State Secretariat for Education, Research and Innovation) and SNSF as one of the leading preventable diseases causing death and disability in the Swiss population.
- Cross-fertilize sepsis and antibiotic/antimicrobial stewardship research.
- Leverage off digitalization for automated data extraction and harmonized data processing using the SPHN interoperability framework and semantics. Explore synergisms across national data streams for the creation of a national sepsis registry.
- Seek partnerships with industry for novel sepsis diagnostics, monitoring, and interventions.
- Develop a strong sepsis patient and public involvement in collaboration with sepsis peer support groups. Prospectively collect at national scale patient and family-reported outcome measures (PROMs).
- Enhance the understanding of the longitudinal trajectories of patients.
- Use multi-omics and large scale high resolution clinical data in collaboration with the ETH domains (PHRT including the Swiss Multi-Omics Center), and SPHN to improve our understanding of sepsis phenotypes across different age groups with the aim to enable more personalized interventions.
- Enhance the effectiveness of sepsis quality improvement by embedded implementation research including health economics.
- Use sepsis as a model disease to build and test a trial platform, which can later be expanded to other diseases and patient groups.

## Discussion

### Public awareness

Sepsis most commonly starts at home. Improved awareness of sepsis is essential to enable timely recognition and intervention which can save lives. Sepsis can affect any member of the society, anytime, anywhere. Therefore, sepsis awareness and education campaigns should be two-tiered: **they must reach the broad population on one side and all healthcare professionals on the other side**. A prerequisite for such multi-level campaigns is consistent terminology and lay wording to make the concept of sepsis widely understandable. A key message is the difference between infection or fever and sepsis – as indicated by signs of organ dysfunction such as difficulties to breathe, poor perfusion, or altered mental state. Sepsis awareness initiatives should thus aim to improve the general health knowledge on sepsis of the population. Such information should include the message that not every infection is sepsis and antibiotics should be reserved for bacterial infections only. Furthermore, public information should help to disseminate information about long-term consequences after sepsis, with different manifestations in different age groups.

Surveys in Germany have failed to identify clear populations in the society which should be primarily targeted – rather, the findings indicate that broad campaigns reaching a high degree of visibility are more effective. Similarly, the UK Sepsis Trust has shifted to advertising in public spaces such as public transport. In New York state, the legislature implemented after the death of Rory Staunton due to sepsis led to a change in the school curricula, demanding that every student is taught on sepsis and signs of sepsis^9^,^10^. In addition, there is a need for sepsis ambassadors in print, audio, television, and social media to spread the information.

Awareness and education campaigns must include healthcare professionals across diverse professions and disciplines; and reach out to both hospital- and community-based professionals. This should lead to a higher awareness, and empower more junior staff, as well as non-medical staff to recognize sepsis early, and to advocate for timely treatment. In Switzerland, pharmacies play an important role as a first point-of-contact and should be included in any effort. In the hospital setting, nurses are often the profession with first, and with most contact with patients and families. Accordingly, the nursing workforce should receive a high priority in sepsis education. Similarly, retirement and nursing home staff, as well as Spitex/CMS (“spitalexterne Hilfe und Pflege,” “Centers Médicaux Sociaux”) care are important areas to include. Importantly, awareness and education campaigns should provide information on long-term sequelae to support the families, and to enable timely recognition of post sepsis syndrome.

For the prevention of sepsis, several existing strategies have been led by FOPH-BAG, and should be further strengthened. Routine vaccinations are highly effective to prevent sepsis (for example, caused by Hemophilus influenzae type B). Vaccinations against influenza for example can reduce the number of cases of sepsis caused to primary viral infection as well as bacterial superinfection of viral infections. COVID-19 vaccinations should serve as an example of the potential of vaccinations to reduce sepsis deaths and morbidity. Similarly, the COVID-19 pandemic has shown that the population can learn to implement simple hygiene measures.

The NOSO strategy outlines efforts to reduce preventable healthcare-associated infections, which is of great importance. Nationally and internationally, extensive literature and materials are available to support effective interventions improving hand hygiene, reducing device-associated infections (such as central line associated blood stream infections (CLABSI), catheter-associated urinary tract infections (CAUTI), ventilator-associated pneumonia (VAP)), as well as reducing wound/postoperative infections. The SSNAP thus strongly recommends to strengthen these activities nationally and locally, in particular those of Swissnoso, to reduce preventable nosocomial sepsis in Switzerland.

### Early detection and treatment

The sepsis severity and mortality, duration of life support, as well as long-term sequelae of sepsis increase with every hour delay to starting appropriate treatment. International guidelines recommend the implementation of systematic screening to assist in timely recognition of sepsis, as well as of institutional protocols to guide sepsis treatment (15, 16). Evidence from case reviews and large observational studies indicates that many patients with sepsis are recognized (too) late; that diagnostic clues to sepsis (clinical or laboratory; such as an increased lactate in septic shock) are often missed; and that, even when sepsis is recognized, there are frequent delays to appropriate treatment and escalation of support. Sepsis thus faces similar problems inherent to the challenge of recognizing sick or deteriorating patients in our healthcare system: there is a gap between ideal (“imagined”) performance of players in a healthcare team (everyone is trained, has time, delivers sufficient attention, and performs at his/her best) and the real world situation (“lived”) where multiple players work together with variable knowledge of the disease, where 24/7 fluctuations of staff presence, seniority, as well as staff workload impose constraints, and where systematic and human barriers are commonly encountered. In order to overcome this gap of compliance with recommended practice, other countries and jurisdictions launched coordinated quality improvement campaigns targeting sepsis (13).

A core component of sustainable sepsis campaigns lies in the definition of a minimal standard for the detection and treatment of sepsis. A standard relates to a bundle of evidence-based principles of clinical management for which a very high compliance is desirable, and which can be measured. Given that sepsis inherently can occur across almost all healthcare specialties, and that sepsis patients may be located in any area of the healthcare system, it is paramount that such a standard is applicable across disciplines, professions, institutions, and regions. That said, healthcare institutions or some of the elements thereof may have particular requirements to fit the patient population they care for – necessitating adaptation of standards to the local context. For example, while every patient with septic shock should receive timely antibiotics, pathways to escalate care may vary locally (ambulance service in a general practice setting; internal ICU for a hospital-based Emergency Department etc.).

In New York state for example, under Rory’s regulations, all hospitals were mandated (i) to have standards for sepsis recognition and treatment in place, (ii) to demonstrate that staff were regularly trained on these, and (iii) to capture sepsis data to allow regular benchmarking and quality control. However, the N.Y. State regulation did not mandate a specific tool or pathway to the hospitals, which allowed institutions to adapt available resources for their local needs. The N.Y. State campaign has been shown to save thousands of lives (31, 32). In the United Kingdom, the UK Sepsis Trust issued the “Sepsis Six” program, outlining key steps for sepsis recognition and treatment almost a decade ago. This allowed a common language and facilitated that different healthcare professions, at all levels of training/experience, could contribute their experience toward better recognition and treatment of sepsis patients.

A key challenge is that the majority of infected patients presenting to healthcare suffer from minor usually self-limiting viral infections and do not develop sepsis-related organ failure. Hence it is essential that approaches to screening and recognition of sepsis focus on “recognizing the sick patient with infection”—i.e. the patient with organ dysfunction, or on a trajectory towards organ dysfunction. While no screening tool is perfect, training, and awareness to recognize presence of cardiovascular dysfunction (shock), respiratory dysfunction (difficulties in breathing, compromised gas exchange), and altered level of consciousness (irritability, lethargy, confusion) are essential. Similarly, while no laboratory marker is perfect, alertness to recognize and respond to laboratory evidence of compromised organ function or tissue hypoperfusion, such as worsening renal function or increased lactate levels, are key. Novel computational approaches can help creation of automatic / digital screening alerts to enhance early detection and for guiding personalized treatment.

At the same time, AMS principles are of paramount importance and should be enhanced through the SSNAP. Specifically, a national standard for sepsis should empower clinicians to “rule-out” sepsis if clinically appropriate, as opposed to “rule-in” sepsis. In many instances, this separation may not be immediately obvious, necessitating a reassessment of the patient and the disease. In addition, effective sepsis treatment resides on appropriate choices and dosing of empiric and targeted antimicrobial therapy. Therefore, sepsis standards should seek to enhance existing local and national guidelines for empiric and targeted antimicrobial therapy, to improve compliance with these, and to ensure contemporary pathogen epidemiology is considered. Finally, a sepsis standard should go hand-in-hand with best practice of AMS, including stopping of antibiotics early if the suspicion of bacterial infection is not sufficiently substantiated, timely consultation with infectious diseases specialists, and streamlining of antimicrobials and their duration depending on infection focus, microbiological results, and severity of disease.

Reliable quality improvement will require robust tracking of the sepsis burden at national level. Previous studies, including national research, have confirmed that using ICD coding will substantially underreport sepsis incidence and burden (33–35).Therefore, a coordinated Sepsis National Action Plan must include a national sepsis registry. In addition to epidemiological surveillance and quality control, a registry will be fundamental for future sepsis research in Switzerland. The registry should build on experiences from existing surveillance databases such as ANRESIS and the Sentinella network, as well as registries such as the Swiss cancer registry. Furthermore, infrastructures created from SPHN/PHRT national datastreams would be ideally suited to support a harmonized data extraction into a sepsis registry. This will allow to create further synergisms and contribute to national pandemic preparedness. As sepsis affects all age groups, it is essential to capture all patients from birth to senescence.

Finally, adherence to the standard in recognizing and treating sepsis, as well as sepsis outcomes should be included in standardized national quality indicators such as ANQ. Separation between community- and hospital-acquired sepsis is key to monitor and target specific interventions. To allow extraction of quality data from hospital data, as well as to improve the quality of the national sepsis registry, training and validation checks of hospital coding for sepsis should be enacted through the existing SwissCode governance.

### Sepsis survivor support

Large observational studies in adults and children indicate that between one in four and one in two sepsis survivors will manifest long-term consequences (4, 36). Long-term effects after sepsis resemble those of post ICU syndrome which has gained attention during the pandemic. Such effects are called “post-sepsis syndrome,” which serves as an umbrella term to characterize the manifold sequelae affecting sepsis. Post-sepsis syndrome includes direct and often life-long physical disability as a result of limb amputation, decreased respiratory capacity after sepsis-associated acute respiratory distress syndrome, or impaired physical activity from combined effects after sepsis. In addition, many patients without obvious physical problems often describe suffering from reduced mental or cognitive capacity after sepsis – survivors often describe that this “invisible” disease has a profound impact on them, leading to much slower recovery than expected, and often being poorly understood by affected patients, families, as well as job contacts. Neonates, children, and adults are all at increased risk of new cognitive impairments after sepsis (37–39). Furthermore, many survivors experience symptoms representing post-traumatic stress disorder, often affecting sleep, relationship patterns, as well as increasing the risk of new or worse mental health problems after sepsis. Altogether, post-sepsis syndrome may decrease educational and professional performance, hinder return to school and work schedules, and impact families as a whole for years to decades to come survivors. Lack of awareness in the broader public as well as by employers may further hinder successful reintegration attempts.

Most healthcare staff such as general practitioners may not be sufficiently aware of the post sepsis syndrome, and patients may not present to them for a structured follow-up. Contrary to myocardial infarction, stroke, or traumatic brain injury, there are rarely well-established follow-up and rehabilitation pathways accessible to sepsis survivors. As a consequence, survivors may miss out on rehabilitation during a window where the adverse long-term effects from sepsis could be mitigated more effectively. In this context, the importance of the transition from hospital to home is essential, with reliable information transfer linking hospital information (such as ICU treatments) with the general practitioner who often represents the primary point of contact after discharge. Furthermore, structured education of allied health services such as physiotherapy and ergotherapy is required to enhance the rehabilitation plan and return to work schedules. Such efforts are likely cost effective, given that indirect costs due to loss of productivity are estimated to exceed direct sepsis costs (8). By consequence, it is imperative that Swiss health insurers consider sepsis follow-up and post-sepsis syndrome as relevant entities, which justify reimbursement of claims related to rehabilitation efforts.

Effective post-sepsis support thus will require a concerted effort which combines education to patients, families, and healthcare staff, with pathways for structured follow-up. This will allow to deploy rehabilitation measures targeted for those most at need. In this context, it is important to address socioeconomic inequities as well as cultural and language barriers – in sepsis, socially more disadvantaged populations may disproportionally suffer from limited access to information, healthcare support, as well as rehabilitation measures.

The widespread impact of sepsis on a family in addition justifies access to professional psychosocial support structures. In addition, professionally assisted peer support groups to assist with debriefing, grieving and loss, and coping strategies are urgently required to support families affected by sepsis. In some instances, such groups may decide to participate in sepsis awareness activities, strengthening the patient and public involvement in sepsis quality improvement to ensure the needs of patients affected by sepsis are met.

### Research

There is an urgent need for better evidence as well as for novel innovative approaches to tackle sepsis as a main contributor to morbidity and mortality in Switzerland. Switzerland, with its high density of academic hospitals, universities, as well as biotech, pharma, and information technology companies, is ideally positioned to drive translational research in sepsis. There are numerous examples of impactful research on sepsis led by Swiss researchers (19), such as the Swiss Pediatric Sepsis Study (18), and the Personalized Swiss Sepsis Study (20). Incentives for sepsis-specific research, such as targeted calls, will be required. Prioritization of pre-clinical and clinical sepsis research at the level of a National Research Program (NRP) is strongly recommended given the huge burden of sepsis on health.

Sepsis-related research should include diagnostic areas of key relevance such as biomarker and biosensor discovery and implementation to improve sepsis recognition. In particular, assisted decision-support systems using artificial intelligence (40, 41) have considerable potential to improve sepsis recognition and early treatment. In addition, the pathophysiology, and the molecular and genetic mechanisms triggering dysregulated host response to infection remain poorly elucidated, providing ample opportunities for basic research. Furthermore, there is a great need for the development and testing of novel interventions such as novel antibiotics and antivirals, as well as testing of highly personalized interventions such as targeted immune therapy. Healthcare service research on the impact and cost effectiveness of quality-of-care programs as well as of innovative diagnostic or therapeutic approaches (such as AI-assisted decision making) is urgently needed. Such should be complemented by qualitative and quantitative evaluation of other implementation aspects including sepsis education to maximize the impact of the SSNAP. Finally, comprehensive research on long-term patient outcomes across different domains of health related quality of life and functional status after sepsis will be essential to develop better approaches to prevent, diagnose, and mitigate the long-term consequences of sepsis.

As evidenced by the COVID-19 pandemic, platform trials capable of testing multiple interventions such as the UK RECOVERY trial, are highly effective and agile means to rapidly mount evidence for best treatments (2). To date, Switzerland has had limited activities in interventional trials in healthcare; investment into investigator-initiated trials, and support for Swiss institutions to participate in international trials is required. Incentives to setup platform trials which can be deployed to answer different key research questions are urgently needed.

Furthermore, effective translation of research into practice, and effective implementation of guidelines into clinical care in the field of sepsis would benefit from structured health service and implementation research to provide high grade evidence on best practice for quality improvement. For this purpose, the availability of a national sepsis database will be paramount, and will enable to target diverse research spanning from health economics to highly personalized interventions. Of note, evidence for optimal rehabilitative interventions after sepsis is scarce. Importantly, the Swiss Personalized Health Network (SPHN) and the Personalized Health-Related Technologies (PHRT) which combine the expertise of hospital, university and ETH domains should promote and support sepsis-specific studies which can build on existing infrastructure such as national data streams. Such will facilitate several key requirements of a comprehensive national sepsis research and quality improvement program, including quality improvement, development and evaluation of novel diagnostic tools, trials on personalized treatment, as well as longitudinal patient trajectories which can capture patient-reported outcome measures (PROM).

Finally, effective patient and public involvement is a prerequisite to drive meaningful sepsis research which will benefit patients, families, and the society. Improving our understanding of the long-term trajectories of sepsis patients through longitudinal studies which elucidate all dimensions of long-term impact after sepsis and will help to delineate the whole-of-life and whole-of-society impact of sepsis.

## Conclusion

Sepsis imposes a major burden to patients, families, the healthcare system, and the society in Switzerland. Although we lack exact current data, estimates based on ICD coding indicate that sepsis affects tens of thousands of Swiss citizens, and is accountable for thousands of deaths and over a billion direct costs in our country every year. The toll of sepsis on human life and societal costs is further multiplied by enormous indirect effects on survivors and families. Yet, Switzerland as one of the wealthiest countries in the world with one of highest *per capita* healthcare expenditures globally, until now has lacked a coordinated approach to reduce the burden of sepsis. It is thus imperative to put this Sepsis National Action Plan into motion, with the view to meet the goals set by the WHO resolution on sepsis in 2017, and the WHO 2030 sepsis plan.

The workshop participants identified the four key themes of awareness and prevention, early recognition and intervention, survivor support, and research as priorities. To address these priorities, the expert panel jointly defined the following key pillars. These pillars relate to strategies which are achievable and can be adapted to the specific Swiss societal and healthcare context:

- **We can learn from others – let us not re-invent the wheel.** While the Swiss setting has unique features, many of the challenges arising around sepsis have been extensively discussed in other countries, who have invested years of expertise in developing and implementing solutions. We have a unique opportunity to approach others such as the Australian Sepsis Network, New York State, the German Sepsis Foundation, or the UK Sepsis Trust, to gain access to expertise, materials, and learn from their lessons learnt. All these healthcare settings have consistently observed that sepsis quality improvement can be immensely effective at reducing deaths due to sepsis through structured, yet relatively simple interventions.
- **Establish a national learning platform to facilitate exchange of resources, data, and materials on sepsis quality improvement**. Swiss Federalism is a reality, and there are many reasons why local healthcare institutions may have to adapt policies and procedures. Yet, this should not block quality improvement in sepsis, nor delay progress in sepsis – a key feature of collaboratives resides in the ability to exchange with colleagues and to learn from each other, while having a common departure base. For this reason, the creation of a multidisciplinary and multiregional Swiss Sepsis Steering Committee is recommended, which oversees several work packages focusing on each of the key recommendations. Such a consensus-focused body would serve to facilitate guidance and exchange of resources and experiences between institutions, while allowing room for each institution to adapt materials to their local needs.
- **Sepsis is an inherently multidisciplinary disease, necessitating broad, integrated approaches**. Sepsis involves many disciplines and groups: families, family doctors, pharmacies, hospitals, insurances, Spitex/CMS, physiotherapy, nursing houses, etc. Sepsis is not “owned” by any specialty, thus necessitating a broader approach reaching out to all areas involved in healthcare.
- **An effective national program against sepsis needs to be interconnected and needs a clear message to the public.** Due to the high interdependencies, it is paramount that a coordinated national sepsis program is simultaneously active across public awareness, healthcare workforce education, prevention, standards of recognition and treatment, data capture and research, as well as long-term survivor support. There is not a single group or a single intervention to prioritize. Sustainability of such a program will rely on all these domains. At the same time, sepsis as a concept remains too little understood and known by the public; and even trained healthcare workers may be insufficiently familiar with sepsis. This places great emphasis on the importance of a professional multilayered public awareness campaign coupled with sustainable educational measures for the broader healthcare workforce.
- **Sepsis is an opportunity to improve the healthcare system, which will benefit many patients - even those who do not have sepsis**. Sepsis is an indicator of the quality of the healthcare system – sepsis is directly affected by aspects such as infection prevention, hand hygiene, choosing wisely components such as central-line bundles, AMS, handovers, speaking up, and interconnected healthcare. Barriers include siloes and fragmented healthcare (institutional, professional, discipline, regional, hierarchical), which will benefit from improved communication, coordination, and setting up of pathways along the patient journey. Sepsis quality improvement thus means improving our health care system. For example, improving the recognition of the septic patient (i.e., the sick/worsening patient with infection) has huge potential to improve the recognition of any deteriorating patient who may benefit from earlier recognition and intervention, even outside sepsis.
- **We can build on existing successful Swiss healthcare programs.** The Swiss HIV campaign, the national vaccination program, Swissnoso, as well as the StAR program on AMS all have demonstrated the benefit of a coordinated national approach to prevent and reduce communicable disease. A Swiss sepsis program should cross-fertilize with these programs. Support and a sepsis specific mandate from federal bodies such as the Federal Quality Commission, and the Federal Office of Public Health is a key requirement for sustainability of such a program.
- **Quality improvement in sepsis means delivering patient-centered medicine.** Tackling sepsis is a chance to improve care with the aim to give patients and families what they want from the healthcare system: better care, faster identification, better outcomes. We can thereby reduce sepsis mortality and improve quality of life of survivors. We can learn from the insights from patients and families to improve our healthcare system; and we can empower them to be active partners in the sepsis prevention, recognition, treatment, and survivor support.

In conclusion, there is urgency to tackle sepsis; we have a unique opportunity to leverage from lessons learnt during the pandemic to address sepsis as the major infection-related threat to our society. With this, we have a responsibility towards our patients, and the society, to commit to effective and evidence-based measures adapted to our country. This will save lives, improve the quality of life of survivors, and reduce the costs for the healthcare system.

## Author contributions

LJS, EAZ, and SMJ oversaw the all work and wrote the first draft. SM, MS, and PMS led the working groups during the workshop. LJS, EAZ, and SMJ refined and developed subsequent manuscript drafts. All authors contributed to the article and approved the submitted version.

## Conflict of interest

The authors declare that the research was conducted in the absence of any commercial or financial relationships that could be construed as a potential conflict of interest.

## Publisher’s note

All claims expressed in this article are solely those of the authors and do not necessarily represent those of their affiliated organizations, or those of the publisher, the editors and the reviewers. Any product that may be evaluated in this article, or claim that may be made by its manufacturer, is not guaranteed or endorsed by the publisher.

## Swiss Sepsis National Action Plan Working Group

Aebersold Daniel; Aebersold Renate; Aebi Christoph, Department of Pediatrics, Inselspital, Bern University Hospital, University of Bern, Bern, Switzerland, 0000–0003–3554-7949; Agyeman Philipp KA, Department of Pediatrics, Inselspital, Bern University Hospital, University of Bern, Bern, Switzerland, 0000–0002–8339-5444; Akrour Rachid, Nursing direction, Lausanne University Hospital, Lausanne, Switzerland; Service of Geriatric Medicine and Geriatric Rehabilitation, Lausanne University Hospital and University of Lausanne, Lausanne, Switzerland, 0000–0001–8248-9089; Albrecht Roland, Swiss Air-Ambulance, Rega (Rettungsflugwacht/Garde Aérienne), Zürich, Switzerland; Berger Christoph, Division of Infectious Diseases and Hospital Epidemiology, University Children’s Hospital Zurich, Zurich, Switzerland, 0000–0002–2373-8804; Bielicki Julia A, Paediatric Infectious Diseases and Infection Prevention and Control, University of Basel Children’s Hospital, Basel, Switzerland, 0000–0002–3902-5489; Borgwardt Karsten, Department of Biosystems Science and Engineering, ETH Zurich, Basel, Switzerland; SIB Swiss Institute of Bioinformatics, Lausanne, Switzerland; SPHN/PHRT consortium on sepsis (www.sepsis-network.ch), 0000–0001–7221-2393; Calandra Thierry, Service of Immunology and Allergy, Department of Medicine & Department of Laboratory Medicine and Pathology, Lausanne University Hospital and University of Lausanne, Lausanne, Switzerland; Caruana Giorgia, Institute of Microbiology, Lausanne University Hospital and University of Lausanne, Lausanne, Switzerland, 0000–0002–2899-1489; Chiche Jean-Daniel, Service of Adult Intensive Care Medicine, Lausanne University Hospital and University of Lausanne, Lausanne, Switzerland, ORCID will be sent; Daniels Ron, Consultant in Intensive Care Medicine, University Hospitals Birmingham NHS Foundation Trust, and Founder and Joint CEO, UK Sepsis Trust; Egli Adrian, Coordination Commission of Clinical Microbiology, Swiss Society of Microbiology, Switzerland; Institute for Medical Microbiology, University of Zurich, Zurich, Switzerland; SPHN/PHRT consortium on sepsis (www.sepsis-network.ch); Ehrhard Simone, Department of Emergency Medicine, Inselspital University Hospital, Bern, Switzerland, 0000–0001–7235-2177; Fellay Jacques, Precision Medicine Unit, Lausanne University Hospital and University of Lausanne, Lausanne, Switzerland, School of Life Sciences, Ecole Polytechnique Fédérale de Lausanne, Lausanne, Switzerland, 0000–0002-8240-939X; Friedrich Marcus, BIH Visiting Professor for the Stiftung Charité; Chief Medical Officer Empire Plan, New York State; UnitedHealthcare; Giannoni Eric, Clinic of Neonatology, Department Mother-Woman-Child, Lausanne University Hospital and University of Lausanne, Switzerland, 0000–0003–0897-6529; Glampedakis Emmanouil, Cantonal Unit for Infection Prevention and Control, Public Health Service, Lausanne, Switzerland; Infectious Diseases Service, Department of Medicine, University Hospital and University of Lausanne, Lausanne, Switzerland, 0000–0002–1920-2265; Glas Michael, Intensive Care Medicine, Emmental Regional Hospital, Burgdorf, Switzerland, 0000–0001–7691-0180; Gouveia Alexandre, Center for Primary Care and Public Health (Unisanté), University of Lausanne, Lausanne, Switzerland, 0000–0002–5798-0092; Grazioli Serge, Division of Neonatal and Pediatric Intensive Care, Department of Pediatrics, Gynecology, and Obstetrics, Children’s Hospital, Geneva University Hospitals and Faculty of Medicine, Geneva, Switzerland, 0000–0002-5526-851X; Haenggi Matthias, Department of Intensive Care Medicine, Inselspital, Bern University Hospital, University of Bern, Bern, Switzerland, 0000–0001-5845-031X; Heininger Ulrich, Infectious Diseases and Vaccinology, University of Basel Children’s Hospital, Basel, Switzerland, 0000–0001–8901-6778; Jakob Stephan M., Department of Intensive Care Medicine, Inselspital, Bern University Hospital, University of Bern, Bern, Switzerland, 0000–0002–2195-0473; Küng Laura; Küng Silvia; Löffel Anton; Marek Roman M., Department of Psychiatry and Psychotherapy, Charité Campus Mitte, Charité -Universitätsmedizin Berlin, Corporate Member of Freie Universität Berlin, Humboldt-Universität zu Berlin, and Berlin Institute of Health, Berlin, Germany; Sepsis Stiftung, Berlin, Germany, 0000–0002–5267-2787; Meylan Sylvain, Infectious Diseases Service, Department of Medicine, Lausanne University Hospital and University of Lausanne, Lausanne, Switzerland; Posfay-Barbe Klara M., Department of Pediatrics, Gynecology & Obstetrics, Pediatric Infectious Disease Unit, Geneva University Hospitals and Faculty of Medicine, Geneva, Switzerland, 0000–001–9464-5704; Pugin Jérôme, Division of Intensive Care, Geneva University Hospitals and Faculty of Medicine, Geneva, Switzerland; Que Yok-Ai, Department of Intensive Care Medicine, Inselspital, Bern University Hospital, University of Bern, Bern, Switzerland, 0000–0001–9443-6101; Rogdo Bjarte, Department of Paediatrics, Cantonal Hospital of Graubünden, Chur, Switzerland; Roger Thierry, Infectious Diseases Service, Department of Medicine, Lausanne University Hospital and University of Lausanne, Lausanne, Switzerland; Schlapbach Luregn J., Department of Intensive Care and Neonatology, and Children’s Research Center, University Children’s Hospital Zurich, Zurich, Switzerland; Child Health Research Centre, The University of Queensland, and Paediatric Intensive Care Unit, Queensland Children’s Hospital, Brisbane, QLD, Australia, 0000–0003–2281-2598; Schwab Patrik, Department of Emergency Medicine, Inselspital, University Hospital Bern, Bern, Switzerland; Sanitaetspolizei Bern, Emergency Medical Service, Bern, Switzerland; Schwappach David, Institute of Social and Preventive Medicine (ISPM), University of Bern, Bern, Switzerland, 0000–0001–8668-3065; Scolari Emil, HESAV School of Health Sciences, HES-SO University of Applied Sciences and Arts, Western Switzerland, Lausanne, Switzerland, 0000–0002–7910-4309; Stocker Martin, Department of Paediatrics; Paediatric and Neonatal Intensive Care Unit, Children’s Hospital Lucerne, Switzerland, 0000–0002-1461-333X; Suter Peter M., University of Geneva, Geneva, Switzerland; Takala Jukka, University of Bern, Bern, Switzerland; Thurnheer Maria Christine, Department of Infectious Diseases, University Hospital Bern, University of Bern, Bern, Switzerland, 0000–0003–1357-0347; Widmer Andreas, Infectious Diseases and Hospital Epidemiology, University Hospital Basel, Basel, Switzerland, 0000–0002–7661-5824; Zimmermann Elisa A., Department of Intensive Care and Neonatology, and Children’s Research Center, University Children’s Hospital Zurich, Zurich, Switzerland, 0000–0002–9958-5912; Zinkernagel Annelies S., Department of Infectious Diseases and Hospital Epidemiology, University Hospital Zurich, University of Zurich, Zurich, Switzerland, 0000–0003–4700-1118.

## Footnotes

^1^ www.krebsliga.ch

^2^ https://www.europeansepsisalliance.org/s/European-Sepsis-Report-FINAL.pdf

^3^ https://www.worldsepsisday.org/declaration

^3^ Federal Office of Public Health FOPH (admin.ch)

^4^ https://www.bag.admin.ch/bag/en/home/strategie-und-politik/nationale-gesundheitsstrategien/strategie-noso--spital--und-pflegeheiminfektionen/ueber-die-strategie.html

^5^ https://www.bag.admin.ch/bag/en/home/strategie-und-politik/nationale-gesundheitsstrategien/nationale-strategie-impfungen-nsi.html

^6^ https://www.bag.admin.ch/bag/en/home/das-bag/publikationen/broschueren/publikationen-uebertragbare-krankheiten/strategie-anbitiotikaresistenzen-schweiz.html

^7^ www.spsp.ch

^8^ https://www.safetyandquality.gov.au/publications-and-resources/resource-library/sepsis-clinical-care-standard-2022

^9^ https://www.nytimes.com/2012/07/12/nyregion/in-rory-stauntons-fight-for-his-life-signs-that-went-unheeded.html

^10^ https://www.endsepsis.org/about-rory-staunton/

## Abbreviations

AMS: Antimicrobial Stewardship
ANQ: Swiss National Association for Quality Development in Hospitals and Clinics
CMS: Centers Médicaux Sociaux
FOPH: Federal Office of Public Health
FQC: Federal Quality Commission
ICD: International Classification of Disease
ICU: Intensive Care Unit
NRP: National Research Program
NVS: National Vaccination Strategy
PHRT: Personalized Health-Related Technologies
PICU: Pediatric Intensive Care Unit
PPI: Patient and Public Involvement
PROM: Patient Reported Outcome Measure
RRT: Rapid Response Team
SERI: State Secretariat for Education, Research and Innovation
SNSF: Swiss National Science Foundation
SPHN: Swiss Personalized Health Network
Spitex: Spitalexterne Hilfe und Pflege
SSNAP: Swiss Sepsis National Action Plan
StAR: Antibiotic Resistance Strategy
WHO: World Health Organization
